# Nondestructive Damage Evaluation in Ceramic Matrix Composites for Aerospace Applications

**DOI:** 10.1155/2013/715945

**Published:** 2013-07-11

**Authors:** Konstantinos G. Dassios, Evangelos Z. Kordatos, Dimitrios G. Aggelis, Theodore E. Matikas

**Affiliations:** ^1^Department of Materials Science & Engineering, University of Ioannina, 45110 Ioannina, Greece; ^2^Department of Mechanics of Materials and Constructions, Vrije Universiteit Brussel, Pleinlaan 2, 1050 Brussels, Belgium

## Abstract

Infrared thermography (IRT) and acoustic emission (AE) are the two major nondestructive methodologies for evaluating damage in ceramic matrix composites (CMCs) for aerospace applications. The two techniques are applied herein to assess and monitor damage formation and evolution in a SiC-fiber reinforced CMC loaded under cyclic and fatigue loading. The paper explains how IRT and AE can be used for the assessment of the material's performance under fatigue. IRT and AE parameters are specifically used for the characterization of the complex damage mechanisms that occur during CMC fracture, and they enable the identification of the micromechanical processes that control material failure, mainly crack formation and propagation. Additionally, these nondestructive parameters help in early prediction of the residual life of the material and in establishing the fatigue limit of materials rapidly and accurately.

## 1. Introduction

Owing to their unique properties such as damage tolerance, fracture toughness, wear and corrosion resistance with respect to monolithic ceramics, and crack growth resistance, CMCs can withstand severe thermomechanical loading conditions [1] and are used today in many aerospace applications as braking systems, structural components, nozzles, and thermal barriers. SiC fibres are the number one candidate reinforcement for such composites as they offer high strength and modulus, thermal stability, and good mechanical performance under high temperatures [2].

The importance of monitoring the structural safety of aerospace structures is imperative. Prevention of catastrophic failure as well as safe and economical management of the structures can be achieved by early assessment of material conditions before the appearance of large-scale fracture. Regular observation of the structures for signs of damage or deterioration will enable the realization of proper repair actions which, in turn, will help extend the useful life span of the component. Among the highly sought-after nondestructive methods capable of monitoring the structural integrity of aerospace structures in an efficient and economical manner, infrared thermography and acoustic emission stand out for being fast, straightforward, and highly reliable. Today both the National Aeronautics and Space Administration (NASA) and Astrium, the European space company, use IRT and AE to detect defects in shuttle wings, rudders and tails, thruster chamber assemblies, and other composite components [3–8]. While IRT captures the thermal energy emissivity of the specimen which is directly related to the damage mechanisms that form and develop during material fracture, the idea behind AE monitoring is that any fracture incident inside a material releases energy which propagates in the form of elastic waves and can be captured at the surface of the material by appropriate sensors. 

While IRT and AE have been successfully applied to detect flaws in CMCs, little information is available on their potential to capture and follow the formation of subsurface cracks. Moreover, it is extremely interesting to investigate the advantages of combined application IRT and AE and to evaluate complementary input that these two techniques can give about CMC damage. 

In the present work, IRT and AE are combined to monitor the formation and development of damage during cyclic and fatigue loading of SiC-fiber reinforced barium osumilite (barium, magnesium, aluminium, and silicate (BMAS)) glass-ceramic matrix composites. IRT was used to identify the most critical, with respect to fracture, damage mechanisms as well as to monitor crack propagation under cyclic and dynamic loads and to predict the composite's residual life. State-of-the-art IR lock-in thermography was used in a unique manner to rapidly and precisely assess the fatigue limit of the CMC, using data from a single specimen test. AE parameters were very powerful in identifying and quantifying real time damage in the CMC. The significance of a large number of IRT and AE indices with respect to mechanical performance and damage evaluation is discussed and explained in the text.

## 2. Experimental

### 2.1. Materials and Mechanical Testing

**Figure 1:**
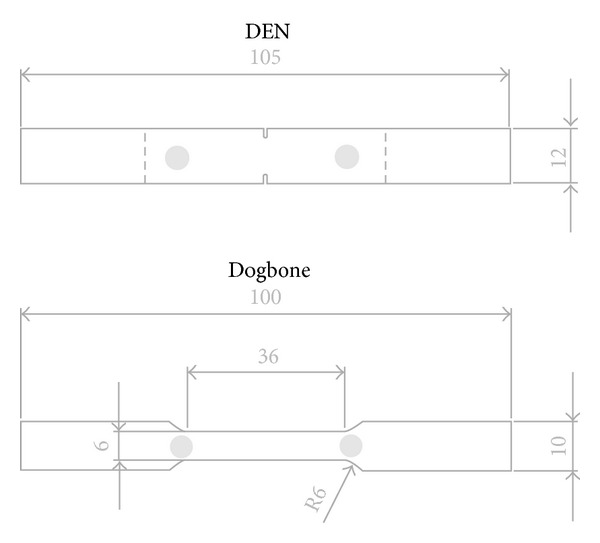
Double-edge-notch and dogbone specimen configurations with marked AE monitoring locations (grey circles).

SiC/BMAS laminates were provided as 3 mm thick plates. The BMAS glass matrix consisted of 50 wt% SiO_2_, 28 wt% Al_2_O_3_, 7 wt% MgO, and 15 wt% BaO and was reinforced by SiC Tyranno fibers stacked and hot-pressed at 1200°C for 10 min in a symmetric (0/90)_4s_ orientation. During hot-pressing, a chemical reaction between the fiber and the oxides of the matrix is known to result in the formation of a weak carbon-rich interphase [9] responsible for large-scale bridging and pull-out phenomena during composite fracture [10]. Rectangular specimens of dimensions 105 × 12 × 3 mm^3^ were prepared in a vertical CNC with fiber orientation in the external plies set to 0° with respect to the specimen's longitudinal axis. Double-edge notch (DEN) specimens of initial notch-to-width ratios of 0, 0.2, and 0.35 were prepared using a diamond wafering blade intended for cyclic tension testing as shown in Figure 1. Dogbone specimens were prepared for monotonic tensile testing as well as for fatigue loading (Figure 1). 

All mechanical testing was performed at ambient temperature on an Instron 8800 servohydraulic frame equipped with a ±100 kN load cell. Specimens were gripped with a pressure of 4 MPa and were tested without end tabs at a nominal gauge length of 50 mm. Static tensile testing, both monotonic and cyclic, was performed under crosshead displacement control with a rate of 0.2 mm/min corresponding to an initial strain rate of 4.0 × 10^−3^ min^−1^ within the 25 mm gauge length of the external, knife-edge-mounted axial extensometer. In cyclic tension experiments with unloading/reloading loops, unloading commenced at 10^−3^ strain and repetitions occurred with a step of 1.5 × 10^−3^ strain. The composites were unloaded to full relaxation before reloading.

**Figure 2:**
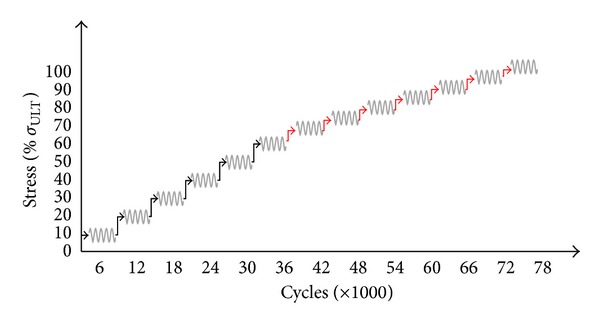
Schematic of fatigue loading protocol.

Fatigue step loading until fracture was conducted on dogbone specimens. The first loading step was set to 10% *σ*_ULT_ and endured for 6000 cycles. The commencing load level was chosen to be low in order to capture the whole mechanical response of the material. The subsequent four loadings, up to 60% *σ*_ULT_, occurred with a step of 10% *σ*_ULT_. At 60% *σ*_ULT_ the fatigue loading step was decreased to 5% *σ*_ULT_ and remained such until specimen fracture. The frequency of the sinusoidal fatigue load was 10 Hz and *R* was 0.1. The fatigue loading protocol is represented schematically in Figure 2.

### 2.2. Acoustic Emission Monitoring

Acoustic emission activity was monitored throughout mechanical testing on all specimens using two “Pico” microminiature AE sensors tape-mounted at a separation of 40 mm on the central part of the specimen (Figure 1). In static tests (monotonic and cyclic tension) the sensors were mounted on the same side of the specimen as the extensometer. This manner of mounting always provided an instrument-free face on the specimen that could be monitored by IRT in real time. Acoustic coupling between the sensors and the specimen was provided by application of silicon grease. The broadband frequency response of the sensors, 50–800 kHz, enabled signal acquisition from a wide range of damage mechanisms. On the other hand, their small size facilitated geometric location of event sources along the specimen. AE sampling was conducted on a PCI-2 board (Physical Acoustics Corporation, Princeton, NJ, USA) with a sampling rate of 5 MHz, an amplification of 40 dB, and a threshold of 45 dB that enabled exclusion of ambient noise from the recorded signal.

### 2.3. Thermography

Throughout testing, temperature variations due to the applied loading, on the AE/extensometer-free face of the specimen, were monitored by an infrared thermography camera (CEDIP, MIW). The camera featured a cooled indium antimonide (InSb) detector (3–5 *μ*m), a focal plane array (FPA) with pixel format of 320 (H) × 240 (V), and a temperature sensitivity of 20 mK. Temperature was recorded with a sampling rate of 100 Hz. Aliasing was avoided by recording the baseline emissivity of the material prior to load application by capturing the IR fingerprint of the surface with the thermal camera. 

Throughout cyclic loading of DEN specimens of the SiC/BMAS composite, thermographs were recorded from the high-stress concentration area between the notches. For lock-in thermography measurements during fatigue loading, specimens were spray-coated with a matte black varnish in order to achieve uniform high-level surface emissivity. Optimal field of view (FOV) conditions were achieved by positioning the camera at approximately 40 cm in front of the gripped specimen. The IR camera was connected to the lock-in amplifier which, in turn, was connected to the servohydraulic controller. This enabled the synchronization of the lock-in amplifier and the testing machine frequencies and capturing of lock-in images and data during fatigue loading. The IR camera was used to measure the amount of energy emitted as infrared radiation, which is a function of the temperature and emissivity of the specimen. According to a previous study, the measured energy corresponds to the intrinsically dissipated energy while the fatigue limit is located at the break of the intrinsic dissipation regime of the loaded specimen [11].

## 3. Results and Discussion

### 3.1. Mechanical Response under Static Tension

**Figure 3:**
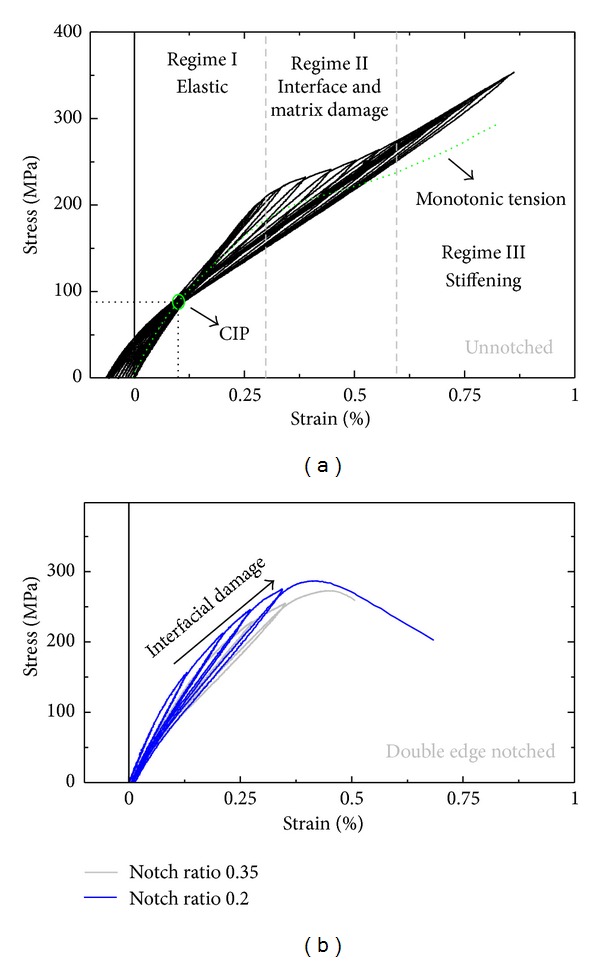
Stress-strain response of SiC/BMAS under monotonic loading (dotted line in (a)) and cyclic tension with unloading/reloading loops for (a) un-notched specimens and (b) notched specimens with various notch lengths.

The stress-strain response of notched and dogbone SiC/BMAS specimens under cyclic and monotonic tension, respectively, is presented in Figure 3. Unnotched specimens exhibited a triple regime behavior consisting of a linear initial part followed by a regime of gradually decreasing tangent modulus and a final regime of apparent stiffening. The second regime (regime “II”) is associated mainly with interfacial damage, most importantly interfacial debonding but also with progressive matrix cracking evidenced as decreasing material stiffness (average slope of unloading/reloading loops). In the third regime (regime “III”) an increase in material stiffness and tangent modulus coupled with an almost linear stress-strain relationship are apparent. In this ultimate regime, the mechanisms of interfacial debonding and matrix cracking have reached a saturated state; hence, material damage is not governed by the interface or matrix anymore; but by a mechanism of superior strength, essentially load bearing by intact fibers [12]. Similar triple regime phenomena with prefailure macroscopic stiffening and linear stress-strain relationships have been encountered before [13]. As observed in the curves of Figure 3(b), Regime III is absent from the mechanical behaviors of notched specimens. This is probably due to premature fiber—hence also composite—stemming from stress concentration in the vicinity of the notch roots. In notched specimens instead, the material failed soon after the maximum load was attained, giving minimal “tail” effects.

Most importantly, the stress-strain curves of unnotched specimens are defined with unique precision, a common intersection point (CIP) of unloading-reloading curves in the first quadrant of the stress-strain curve in the tension domain. The coordinates of the CIP, 0.001 strain and 90 MPa stress, are directly related to the axial residual stress state of the composite [14, 15]. While a thorough analysis of the CIP feature for the particular composite has been the subject of a previous work [16], it is interesting to repeat here that a self-assembled CIP had never before been encountered experimentally. 

Comparing the monotonic and cyclic tension curves for the SiC/BMAS composite (Figure 3(a)), it can be concluded that cyclic loading results in an increase by 20% in attainable material stress, calculated at fracture. If this increase is due to higher amounts of energy dissipated at damage mechanisms such as interfacial debonding, matrix cracking, and load bearing by intact fibers [10], it is then suggested that cyclic loading by itself improves the energy dissipation capacity of the material. The existence of another energy dissipation mechanism, pull out, anticipated by the weak interfacial bond discussed in the experimental section, was verified after the end of the tests: failed specimens had not separated in two pieces after removal from the grips, with the frame still indicating small load values of the order of a few Newtons. This meant that fibers had failed within the matrix environment and had pulled out noncompletely before removal of the specimens from the grips [17]. 

Composite strength and modulus appeared to increase with decreasing notch length. Unnotched specimens enjoyed average strengths and moduli of 355 MPa and 151 GPa, respectively. The corresponding values for the 0.2 and 0.35 notched-to-width length were 280 MPa/119 GPa and 270 MPa/108 GPa, respectively.

### 3.2. Thermography

#### 3.2.1. Static/Cyclic Loading

**Figure 4:**
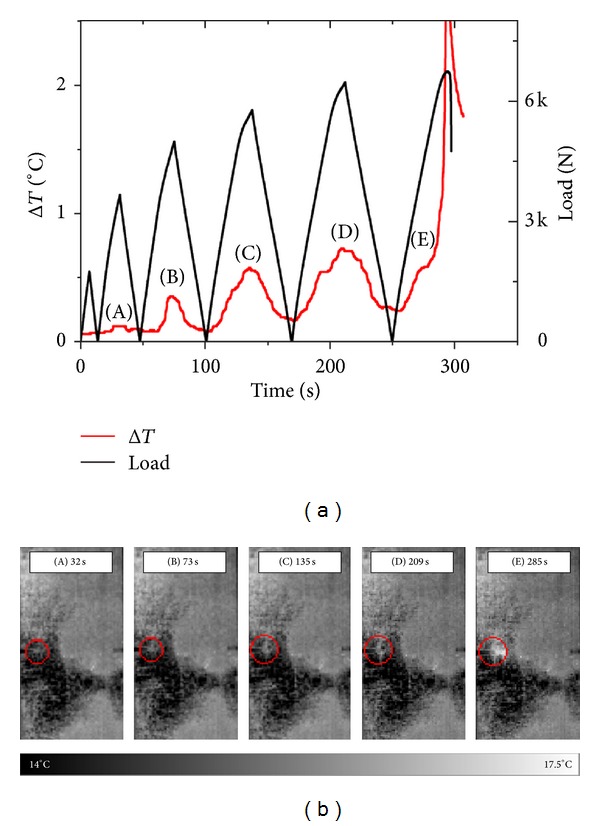
(a) Δ*T* and load versus time for a 0.35 notch-to-width ratio specimen loaded in cyclic tension. Sequential alphabet letters indicate instance of thermographs presented in (b).

Temperature variation as measured by IRT, Δ*T*, and load is shown in Figure 4(a) as a function of time for a DEN specimen with a 0.35 notch-to-width ratio. Indices “A” to “E” denote the instances of the thermographs shown in Figure 4(b), collected at the notched ligaments of the composites. It is observed that peak Δ*T* location coincides with the location of maximum load for every cycle, whereas peak Δ*T* magnitude increases with progressing loading, hence also material damage. At the ultimate cycle, the Δ*T* trace appears to follow a completely different pattern than those in previous cycles, wherein temperature appears to drastically increase, indicating that the specimen is heading for catastrophic fracture.

In Figure 4(b), the locations of crack initiation, as identified by IRT, are indicated by a red circle mark. The apparent high-temperature area located outside and to the right of the circle mark is a baseline pattern that exists even before load application and remains constant until catastrophic failure. It is associated with the specimen's surface emissivity, not with material damage. It should not be ignored that IRT is concerned with temperature* variations* as a result of progressive damage, not with *absolute* values. Under this rationale, no noticeable change in temperature is seen up to 32 sec experimental time (thermograph (A) of Figure 4(b)). 73 sec within testing (thermograph (B) of Figure 4(b)), very small temperature variations can be observed within the marked (circle) area. Temperature increases become more obvious in thermographs (C) and (D) of Figure 4(b), 135 and 209 into loading, respectively. It is indicated that the damage is extending in area and magnitude. In the last loading cycle, 285 sec in the test, a dramatic increase in temperature throughout the whole notched ligament signifies that material failure is imminent.

**Figure 5:**
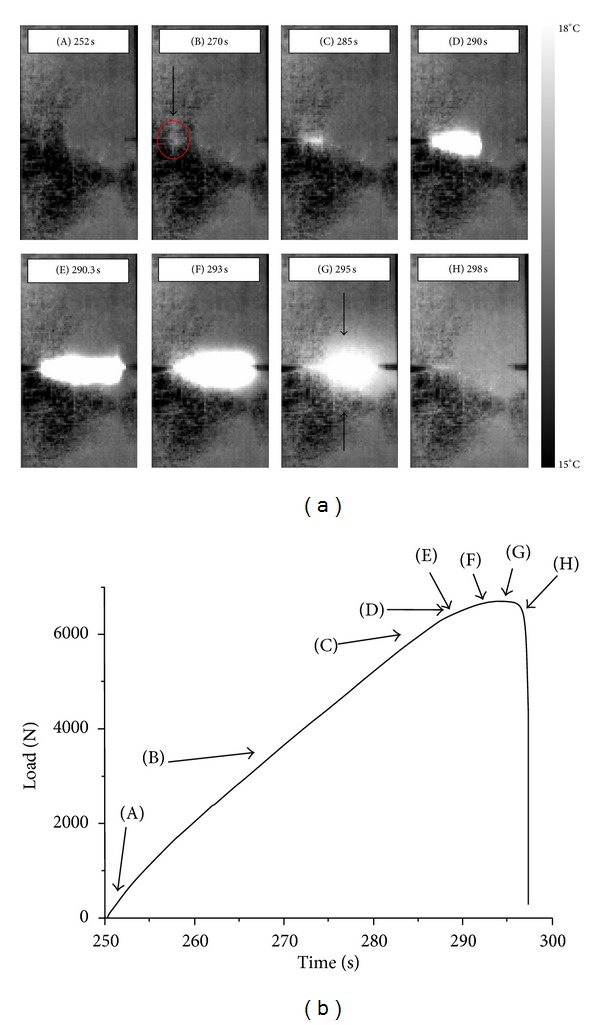
Final loading cycle of 0.35 notch-to-width ratio DEN specimen. (a) Thermographs showing crack propagation and (b) load versus time curve.

The thermographic behaviour within the ultimate loading cycle, of the same 0.35 notch-to-width ratio specimen, is demonstrated in Figure 5 and analyzed in the following. Indices “A” to “H” presented in Figure 5(b) define the instances of the thermographs in Figure 5(a) (note the notch roots). In thermograph (A) of Figure 5(a), no hot appears exist within the notched ligament. In the next instance, thermograph (B) of Figure 5(a), a red arrow indicated what appears to be crack initiation. At 285 sec, thermograph (C) of Figure 5(a), a significant temperature difference is observed, which coincides with the change in the slope of the mechanical response curve. It is believed that from this instance on, subsurface crack starts propagating from the left notch root with direction to the right. It is important to establish this instance as precisely as possible, as this will facilitate early prediction of the final fracture. It is noted that the associated time (285 sec) corresponds to 73% of the total duration of this ultimate loading cycle. 5 sec later (thermograph (D) of Figure 5(a) at 290 sec) the subsurface crack appears to span half of the notched ligament while only another 300 msec later (thermograph (E) of Figure 5(a)) it propagates abruptly and unstably towards the right notch. The maximum temperature is attained (thermograph (F) of Figure 5(a)) at an instance that coincides with the maximum load of the final loading cycle. This temperature is associated with the matrix cracking saturation and the load bearing completely by the reinforcing fibers. Temperature starts decreasing at the left notch in the next thermograph, (G) of Figure 5(a), while it increases above and under the subsurface crack, as indicated by black arrows. This increase is due to the fiber failure under the critical level of applied load. Failed fibers pull out giving rise to the frictional thermal energy evidenced in thermograph (H) of Figure 5(a). 

**Figure 6:**
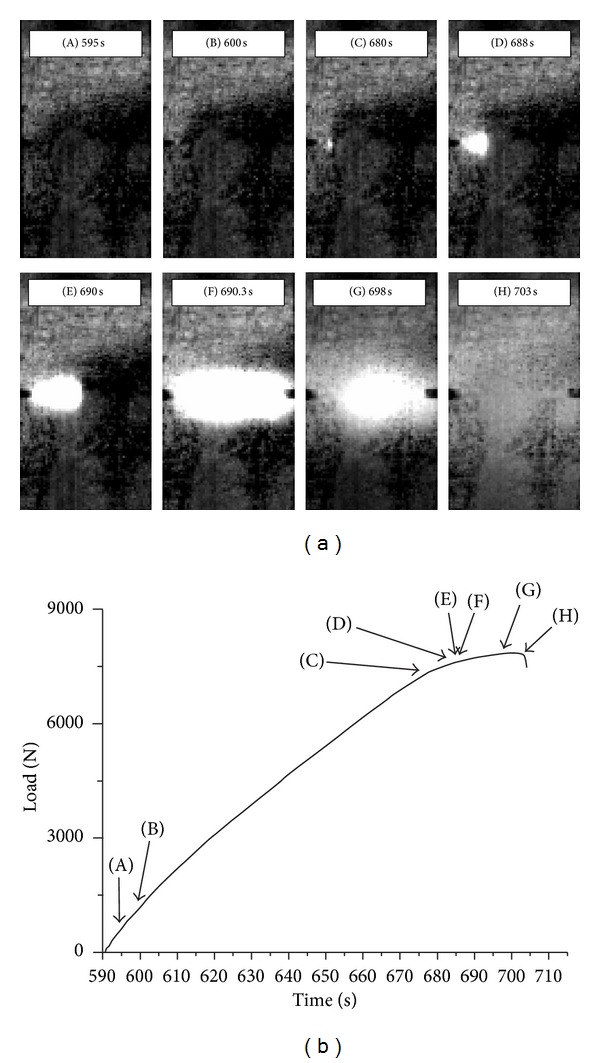
((a), (b)) Thermographs of crack propagation and diagram of load versus time of final loading cycle (0.2 notch-to-width ratio specimen).

Similar trends were observed for DEN composites with smaller notches, as in specimens with 0.2 notch-to-width ratios; the thermographic behaviour within the ultimate loading cycle of such a specimen is demonstrated in Figure 6 and analyzed in the following. Again, indices “A” to “H” presented in Figure 6(b) define the instances of the thermographs in Figure 6(a). After 600 sec of testing time, Figure 6(a)(B), no “warm” damage areas are seen in the thermographs. Crack initiation appears at 680 sec, Figure 6(a)(C), which compares favorably with the instance of slope change in mechanical behavior of the material. It is hence possible to foresee early fracture still at 80% of the final cycle duration. In the subsequent thermographs Figure 6(a)(D) and (E), the subsurface crack propagates from the left notch towards the middle of the notched ligament. The crack then propagates abruptly and unstably towards the right notch. A temperature variation profile compatible with pull out is seen again in the last thermograph, Figure 6(a)(H), where the specimen has failed completely.

**Figure 7:**
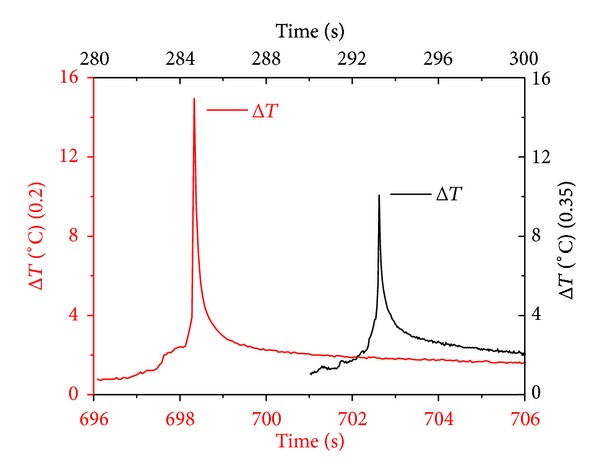
Peak Δ*T* for the two notch lengths used in the DEN specimens.

Peak Δ*T* is shown in Figure 7 for the two notch lengths used in the current study. It is observed that specimens with shorter notches exhibit Δ*T* of 15°C at fracture while the ones with longer notches exhibit Δ*T* values around 10°C. It is believed that in specimens with larger notched ligaments (smaller notches), damage evolves over a wider material region throughout testing; hence peak temperature at the critical load is not high. On the other hand, damage is accumulated and relieved not so drastically in a specimen with less material available within the notched region.

#### 3.2.2. Fatigue

**Figure 8:**
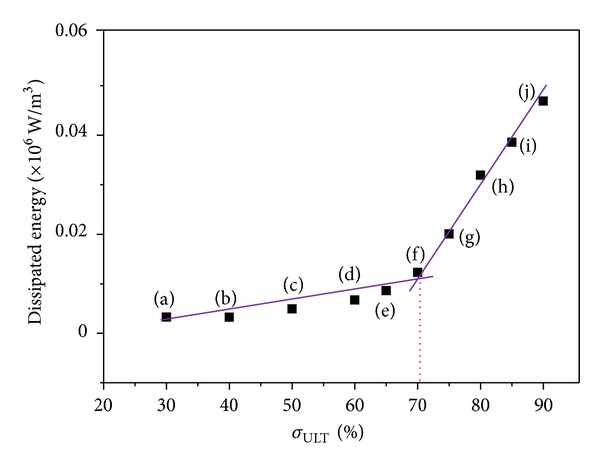
Dissipated energy versus % *σ*_ULT_ for fatigue-loaded SiC/BMAS.

Lock-in thermography was applied during fatigue loading of SiC/BMAS dogbone specimen. The intrinsically dissipated energy as monitored by the IR camera for 10 different stress levels ranging from 30% to 90% *σ*_ULT_ is plotted as a function of % *σ*_ULT_ in Figure 8. 

The curve exhibits two distinct slopes, visualized by the two linear regressions seen in Figure 8. At low stress levels, 30% to 60% *σ*_ULT_, the dissipated energy increases with a low rate, while from 70% *σ*_ULT_ and upwards it rises considerably more rapidly. The intersection point of the two lines defines the fatigue limit of the material. The value of fatigue limit calculated for the cross-ply SiC/BMAS through the thermographic approach of this study is 70% *σ*_ULT_ or 205 MPa.

**Figure 9:**
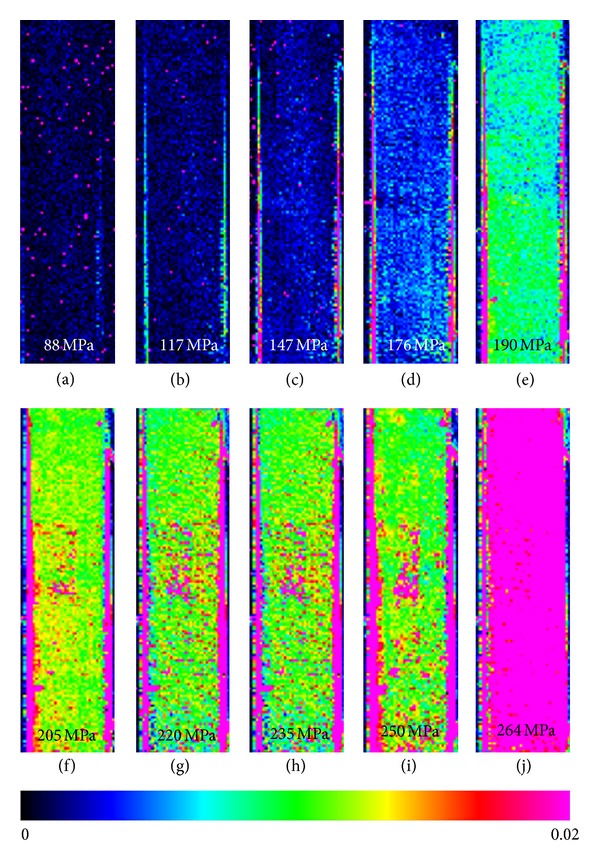
Thermographic pattern during fatigue loading of a SiC/BMAS composite.

An examination of the thermographic pattern of cross-ply SiC/BMAS at different stress levels is of particular interest in view of the established fatigue limit value. This information is presented in Figures 9(a)–9(j), wherein the 70% *σ*_ULT_ fatigue level which corresponds to thermograph Figure 9(f). Two distinct cases are made obvious by examination of this figure: (i) thermographs Figures 9(a)–9(d) depict low energies in cold (blue) color coding in the initial loading stages associated with minimal material damage and (ii) thermographs Figures 9(e)–9(j) capture progressive damage accumulation which is captured by increasingly warmer colors (high energy). In the first four thermographs, up to 176 MPa applied stress, there is practically no appreciable change in the dissipated energy. In the fourth thermograph, Figure 9(e), a slight change in color can be attributed to the saturation of elastic energy accumulation on the onset of appearance of fatigue. A totally dissimilar energy distribution pattern appears in thermograph Figure 9(f) due to the unfolding of internal energy dissipation phenomena such as interfacial damage, delamination, and fiber sliding across the debonded interface [18]. At thermographs Figures 9(g)–9(j) (75%–90% of *σ*_ULT_), a raise in energy can be noticed indicated by the increment of the magenta spots until fracture. At the particular loading level, this energy can be attributed to fiber bridging, fiber failure, and pull out.

### 3.3. Acoustic Emission

#### 3.3.1. Static/Cyclic Loading

**Figure 10:**
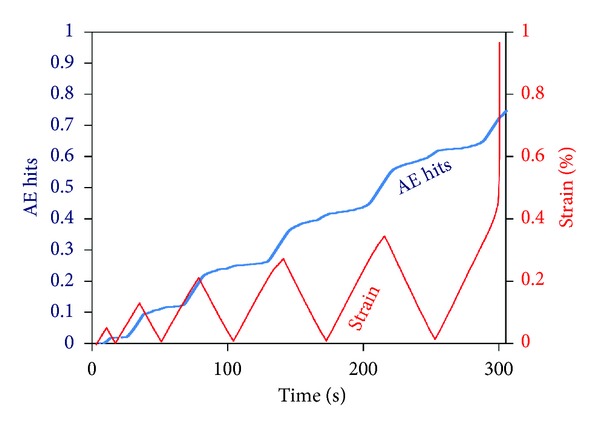
Strain and AE cumulative history for specimen B.

Cumulative AE signal history collected during cyclic loading of a DEN specimen with 0.35 notch-to-width ratio is shown in Figure 10 alongside with strain. The rate of AE acquisition exhibits fluctuations according to the cyclic loading protocol. Specifically, AE rate increases as the load increases to the maximum within each cycle. After these maxima points, the AE rate decreases without however being completely eliminated, at the cycle's minimum load. The total activity was of the order of 4000 signals.

Apart from the cumulative activity, which counts the separate acquisitions of the sensors, different AE descriptors help to distinguish the severity of the condition according to loading level. Two of them are the ASL and RMS. ASL is the average signal level defined as the average amplitude of samples of the rectified waveform while RMS is the square root of the average of the squares of all points of a waveform (root mean square) [19].

They are given by

(1) ASL  (Average  Signal  Level):  ASL=1n(x1  +x2  +⋯+xn  )RMS  (root mean square,Xrms):  Xrms=1n(x12+x22+⋯+xn2),

where *n* is the number of samples (waveform points) and *x*_*i*_ stands for the rectified amplitude of the waveform samples.

**Figure 11:**
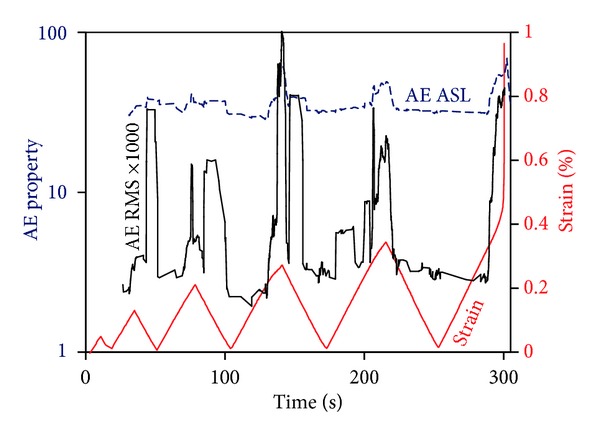
Strain history and AE amplitude parameters for a double-edge notched specimen with notch-to-width ratio of 0.35.

Therefore, both of them are indicative of the AE signal emitted by the fracture.  Figure 11 shows the sliding average (window of 20 points) for both AE parameters. Focusing on the ASL first, local peaks are indicated at the moments of local maxima of strain, especially for the last three cycles. This means that at those periods of high strain, stronger fracture incidents take place which are recorded as waveforms with different intensity characteristics. The same holds for the RMS which again gives a measure of the elastic energy recorded by the sensor being proportional of the pressure wave that impinged on the sensor's surface. The values of RMS were multiplied by a thousand for visibility reasons in the graph. These parameters show that monitoring of AE and specific qualitative AE features allows the recording of the high stressing moments and the whole loading history until the material is brought to failure.

**Figure 12:**
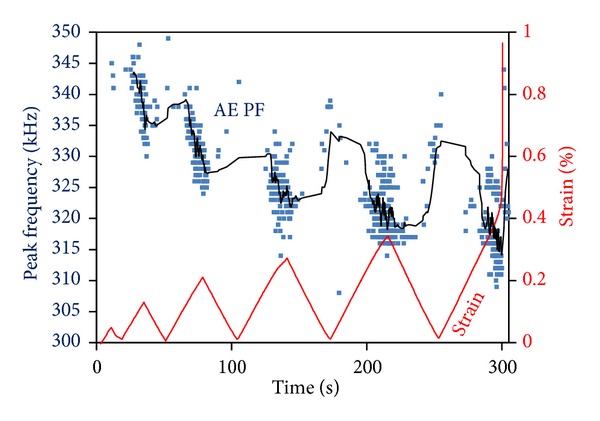
Strain history and AE peak frequency for a double-edge notched specimen with notch-to-width ratio of 0.35. The solid line is the sliding average of recent 20 points.

Apart from the amplitude or energy-related parameters, significant information can be derived by the frequency content of the emitted waves. In mechanical materials it has been shown that a drop of frequency indicates increase of damage accumulation and is linked with the shift between fracture modes (e.g., initial tensile matrix cracking to ultimate shearing) [20, 21]. The index that is displayed here is peak frequency (PF) and is the frequency of the highest peak of the FFT of the AE waveforms. Using the specific AE sensors typical peak frequencies average around 450 kHz which is the maximum sensitivity of the sensors, while different bands from 100 kHz up to approximately 800 kHz can also be recorded.  Figure 12 focuses on a specific family of emissions which initially exhibit frequencies between 330 and 350 kHz. It can be easily seen that this group of emissions are registered near the maxima of the strain cycles. However, one certain trend is that the main frequency of this family steadily drops in frequency after each cycle. This drops is of the order of 5 to 10 kHz in each cycle resulting in an average of 315 kHz at the last cycle just before failure. This drop of frequency is a result of the shifting between fracture mechanisms within the material since the monitoring conditions (sensors, separation distance) were constant throughout the experiment duration. 

**Figure 13:**
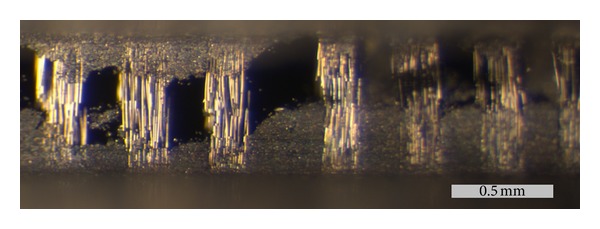
Stereoscope image of the side of a fractured specimen.

The observed behavior can be due to the increasing number of interfacial F that is expected to happen at the higher strain levels of the cycles. This allegation is supported by visual evidence of fiber bundle sliding and existence of off-axis layers seen in the postmortem side view of a specimen's notched ligament, microphotograph in Figure 13. It is observed that crack opening is approximately 500 *μ*m. It is also observed that fibers are bridging the crack sides being pulled out of the matrix. While rupture of a large number of individual fibers is evident, fiber bundles have not failed completely since the specimen was removed in one piece after the test. 

#### 3.3.2. Fatigue Loading

**Figure 14:**
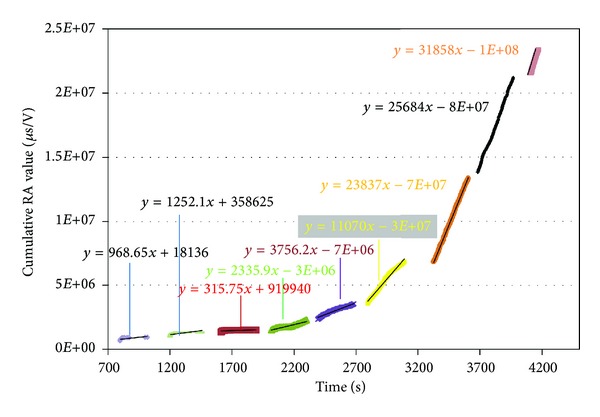
Cumulative RA value for the different fatigue loading stages.

During fatigue loading continuous monitoring by AE was applied, as mentioned earlier. Apart from the activity (number of emissions) all different AE parameters were recorded. As the fatigue life proceeds and damage is being accumulated, apart from the larger number of signals which are emitted, the nature (waveform shape) of the AE incidents starts to change. One of the indicative AE descriptors is the RA value which is the inverse of the rising angle of the waveform and is given by

(2) $$\begin{array}{c} \text{RA}=\frac{\text{RT}}{A}, \end{array}$$

where RT is the rise time of the waveform (or delay between the onset and the highest peak in *μ*s) and *A* is the amplitude of the highest peak in V. In general this feature increases as damage develops along with the shift of the failure mechanisms from matrix cracking to shearing phenomena like interfacial sliding and debonding [18, 19]. Therefore, RA values are progressively increasing until the final failure of the specimen.  Figure 14 shows the cumulative RA value for the different stages of fatigue loading. As mentioned above, at the early stages, RA values accumulation is low. At the block of loading starting at approximately 2000 s (70% of UTS) the RA values seem to accumulate at a higher rate, which is increasing for each successive step. 

**Figure 15:**
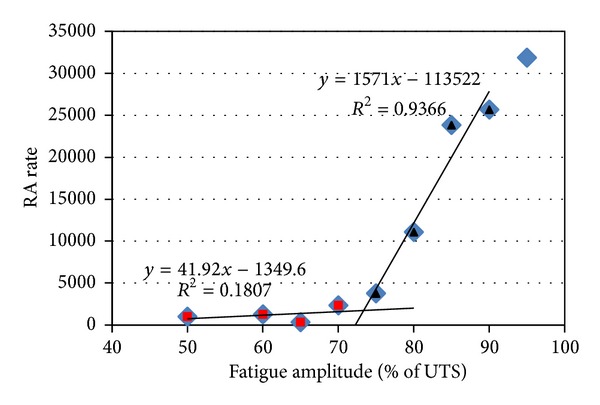
RA increasing rate for different fatigue loading amplitudes.

The rate of RA accumulation is shown in Figure 15. This allows a more clear visualization of the trends between the AE and RA at different stages. It is concluded that the curve can be fitted by two straight lines. The change of slope between the two lines occurs below 75% load which is a certain indication that significant changes take place after that load level. At lower loads, the events emit notably lower RA values showing less intensity compared to levels higher than 75%. The intersection of the two lines is between 70% and 75% and coincides well with the change of slope in the corresponding thermal dissipation curve presented earlier. This shows that both techniques can register small but delicate changes of the failure processes, depending on the fatigue loading. It seems that although incidents that, emit acoustic emission and heat are induced by low loading, these exhibit distinct values from corresponding events at loads higher than the fatigue limit which eventually will lead to the failure of the specimen. 

## 4. Conclusions

Thermography and acoustic emission were used to capture the initiation and evolution of damage in SiC-fiber reinforced glass-ceramic matrix composites under static and fatigue loading. Infrared thermography results helped identify the intact fiber population as the mechanism that control ultimate material failure and that under the presence of notches the composite fails shortly after the attainment of a saturated matrix cracking state. Infrared thermography (IRT) was also used to monitor, both in location and in time, the crack propagation path during mechanical testing in cyclic tension and fatigue. The technique also enabled early prediction of the residual life of the material, as early as at 73% of the duration of the final loading cycle. Successful application of the technique under such dynamic conditions where the surface changes with usage is close to real-life scenarios found in aerospace applications. 

A novel infrared lock-in thermographic methodology was used for the determination of the fatigue limit of the ceramic matrix composites (CMCs). The limit was unconventionally rapidly assessed by the thermographic technique at 70% *σ*_ULT_ (i.e., 205 MPa). The outcome makes lock-in IRT a new, versatile, and accurate method that overcomes the limitations of Wöhler's curve approach, as it significantly reduces experimental time and requires testing of a single sample only for obtaining the fatigue limit of the material.

Furthermore, acoustic emission (AE) monitoring enables monitoring fracture behavior in real time. Apart from the increase of AE acquisition for higher load and damage accumulation, energy- and frequency-related parameters help discern the moments higher stress. Descriptors like the root mean square (RMS) and average signal level (ASL) increase their values at high stresses, while peak frequency shows the inverse trend, being continuously downgraded for the successive loading steps. Concerning fatigue, AE showed the capability of detecting the different intensity of the fracture incidents. Waveform shape parameters like the RA exhibit changes as the load increases above the fatigue level of the material. The results are benchmarked by the heat dissipation curves offered by thermography and allow the determination of the fatigue limit of the material by using only one specimen.
